# Roughness of the Surface of Zirconia Reinforced Lithium Disilicate Ceramic Treated by Different Procedures

**DOI:** 10.3390/ma16010265

**Published:** 2022-12-27

**Authors:** Andreja Carek, Ljerka Slokar Benić, Daniel Komar, Ena Krebelj

**Affiliations:** 1 School of Dental Medicine, University of Zagreb, Ivana Gundulića 5, 10000 Zagreb, Croatia; 2 Faculty of Metallurgy, University of Zagreb, Aleja narodnih heroja 3, 44000 Sisak, Croatia

**Keywords:** laser irradiation, surface treatment, surface roughness, zirconia reinforced lithium disilicate, self-adhesive resin cement

## Abstract

Lithium disilicate and zirconia are the two most popular materials for aesthetic and dental prosthetic work; however, due to their limitations, a new material is being researched, namely zirconia-reinforced lithium disilicate, the surface of which is treated with different procedures to achieve the best possible surface properties. In this study, the surface of zirconia-reinforced lithium disilicate glass-ceramic was treated using different methods (conventional and laser) to determine the effects of the treatment procedures on the surface properties and surface roughness to achieve a higher strength of adhesion from the self-adhesive resin cement to zirconia-reinforced lithium disilicate. The treated surfaces were investigated using profilometry, X-ray diffraction and energy dispersive X-ray fluorescence. The results obtained were statistically evaluated. The results show that the surface roughness is highest for the samples treated with Er:YAG (erbium-doped yttrium aluminium garnet laser) and silanisation. Furthermore, the surface treatment procedures applied did not change the composition of the surface.

## 1. Introduction

Due to increasing patient demand for more aesthetic and tooth-like prosthetic work, metal-ceramic works were no longer desirable, which led to the development of all-ceramic materials. Among the most popular materials, lithium disilicate (LDS) and zirconia are used, but both have their limitations [1]. Lithium disilicate (LDS) has excellent aesthetic properties, especially translucency, but its mechanical properties cannot meet the requirements of the molar region for dental bridges [2]. On the other hand, zirconia (ZrO_2_) is known for its high flexural strength of 840–1200 MPa (LDS shows 370–460 MPa), making it suitable for crowns and bridges in the aforementioned posterior region [3]. From a desire to combine the best of these two worlds, lithium disilicate glass-ceramic has been reinforced with zirconia. Zirconia-reinforced lithium silicate (ZLS) is a material that has been available in the form of blocks for CAD/CAM (computer-aided design/computer-aided manufacturing) fabrication since 2013. It was developed by two German companies, Vita (Vita Zahnfabrik, H. Rauter GmbH & Co., Bad Säckingen, Germany) and Dentsply (Dentsply Sirona, DeguDent, GmbH, Hanau-Wolfgang, Germany) [4]. The special feature of ZLS is the good combination of aesthetics and mechanical properties, which makes it a possible restorative material for both anterior and posterior teeth. The aesthetics are achieved by a high proportion of glassy matrix, while the mechanical properties are improved by zirconia in a proportion of 8–12% [5]. The ZLS contains a specific microstructure: very fine lithium metasilicate, disilicate and orthophosphate crystals, and a glassy matrix with tetragonal zirconia. The aforementioned microstructure enables high flexural strength with good optical properties and makes it easy to polish. In addition, ZLS has a higher flexural strength, modulus of elasticity, hardness and fracture resistance compared to conventional leucite-reinforced glass-ceramics. The flexural strength of ZLS ranges from 405 to 553 MPa, which makes ZLS the strongest material among glass ceramics [6]. Furthermore, unlike zirconium oxide, ZLS can be etched and cemented with adhesive systems. This is another advantage of ZLS, as the fracture resistance of adhesively cemented crowns is higher than that of conventionally cemented ones [7].

Whether the ZLS has a higher composite bond strength than the LDS remains to be explored. Fonzar et al. reported that ZLS has a higher bond strength after etching than LDS [8], while Zhang et al. found that its bond strength to resin composites is similar [9]. ZLS fabricated with CAD/CAM is used for inlays, onlays, veneers, anterior and posterior crowns and single-tooth restorations on implant abutments [1,3,10]. The advantage of CAD/CAM fabrication is the simplified manufacturing process. This ensures less risk of deformation of the dental prosthesis, which often occurs with conventional procedures due to their complex and numerous steps [11]. The number of appointments is also reduced and the fabrication time is shorter, resulting in less tooth movement. This makes the restoration of the tooth less traumatic and more precise [12]. As mentioned earlier, the importance of ZLS lies in the combination of good optical and mechanical properties. Cementation is required to secure the fixed prosthetic work, which provides retention and stability in three ways: mechanical retention, micromechanical bonding and molecular adhesion. Micromechanical bonding depends on surface roughness. Surface texture consists of the tiny irregularities in the form of bumps and dimples and is composed of two components—undulations and roughness [13]. The roughening of the surface enables interlocking between ZLS and resin cement, which increases adhesion [14]. Surface roughness can be achieved by various surface treatments, which can be divided into conventional and laser surface preparation protocols. Conventional protocols include hydrofluoric acid (HF) etching, sandblasting, silanisation and combinations thereof, while laser protocols use Nd:YAG (neodymium-doped yttrium aluminum garnet) and Er:YAG lasers. Etching and silanisation were considered the “gold standard” for bonding composite cement to ceramic, as the acid HF creates a mechanical bond and the silane creates a chemical bond [7]. Laser irradiation is considered a modern method for treating ceramic surfaces. Lasers concentrate and focus an enormous amount of energy on a small target area, which causes micromorphological physical changes by absorbing the energy [15]. Sevmez and Yilmaz demonstrated that different surface treatments have different effects on surface roughness and bond strength in ZLS, but also that higher surface roughness does not always lead to higher bond strength [14]. Dalla-Nora F et al. reported that tribochemical silica coating (air-particle abrasion) to treat the ZLS surface before bonding should be avoided as it leads to poorer fatigue results, while HF acid and self-etching primers improve mechanical fatigue behaviour [16]. Komar et al. concluded that surface treatment by Nd:YAG irradiation improves the bond strength of resin cement to ZLS compared to conventional protocols [7].

The aim of this study is to investigate how different surface treatment procedures can influence the surface roughness of ZLS.

## 2. Materials and Methods

Lithium disilicate glass-ceramic reinforced with zirconia (Suprinity, Vita Zahnfabrik, Bad Sackingen, Germany) was used for this study. CAD/CAM (computer-aided design/computer-aided manufacturing) blocks were cut in pre-crystallized form on 70 samples with dimensions 18 × 12 × 2 mm. Cutting was performed with the Isomet 1000 cutter, followed by crystallisation in the Programat P300 furnace (Ivoclar Vivadent AG, Schaan, Liechtenstein). To be prepared for polishing, the samples were then mounted in a silicone mould. Polishing was conducted on a grinding/polishing machine equipped with a Vector LC power head and sample holder (Phoenix Beta, Buehler, Germany). Polishing was conducted with 600 grit sandpaper for one minute per sample at 300 rpm. After polishing, the samples were embedded in acrylic and their position was completely fixed. After all the samples were prepared, they were divided into 7 groups (A-G) according to the surface treatment procedures (Table 1). 

The samples in the control group (A) were not treated further after polishing and fixing. The surface of the samples in group B was treated with 9.5% hydrofluoric acid (Bisco Inc., Schaumburg, IL, USA) for 90 s. The samples were then washed and dried according to the manufacturer’s instructions. The surface of the samples in group C was treated with silane (Monobond Plus, Ivoclar Vivadent AG, Schaan, Liechtenstein), which was rubbed in with a brush for 1 min. Group D represents a combination of the treatments of groups B and C. The samples were first treated with 9.5% hydrofluoric acid (Bisco Inc., Schaumburg, IL, USA) for 90 s (then washed out and dried), then the surface was rubbed with silane (Monobond Plus, Ivoclar Vivadent AG, Schaan, Liechtenstein) with a brush for 1 min. The surface of the samples in group E was sandblasted with Al2O3 particles of 30 μm size (Co-Jet Sand, 3M ESPE, Neuss, Germany) using a Renfert Basic Master sandblaster (Renfert dental, Hilzingen, Germany). Sandblasting lasted 15 s at a pressure of 2.7 atm. The sandblaster was 1 cm away from the sample in the vertical direction. Then, the samples were washed, dried and blown off to remove residual particles. They were then silanised by rubbing silane (Monobond Plus, Ivoclar Vivadent AG, Schaan, Liechtenstein) onto the surface of the samples with a brush for 1 min. The surface of the samples in group F was treated with Er:YAG irradiation (LightWalker, Fotona, Slovenia) with a pulse energy of 500 mJ, a power of 10 W and a frequency of 4 Hz for 20 s. Silane (Monobond Plus, Ivoclar Vivadent AG, Schaan, Liechtenstein) was then rubbed onto the surface of the samples with a brush for 1 min. The surface of the samples in group G was treated with a Nd:YAG laser (LightWalker, Fotona, Slovenia). The parameters used are a pulse duration of 100 mJ, a frequency of 20 Hz and a power of 1 W. After laser irradiation, silane (Monobond Plus, Ivoclar Vivadent AG, Schaan, Liechtenstein) was rubbed onto the surface of the samples with a brush for 1 min. All surface treatment procedures were carried out according to the manufacturer’s instructions. After surface treatments, the samples were subjected to surface roughness analysis, as well as XRD (X-ray diffraction analysis) and EDXRF (energy dispersive X-ray fluorescence) analysis. 

All surface roughness measurements were performed at the National Laboratory for Length at the University of Zagreb. Surface roughness was measured according to ISO 4287:1997 [17], ISO 4288:1996 [18] and ISO 3274:1996 [19] using the contact stylus 2D profile method with the Perthometer S8P (Feinprüf Perthen GmbH, Göttingen, Germany). The roughness was analysed on the first, third and fifth sample of each group. Six roughness profiles were measured on each sample. A Gaussian philtre with a cut-off value of λc = 0.8 mm, a probe radius (r) of 5 μm, a measuring force of 1.3 mN, an evaluation length (ln) of 4.0 mm and scan length of 5.6 mm was used. 

X-ray diffraction (XRD) analysis was performed to determine the mineral composition of the samples. X-ray diffraction analysis was performed using the Shimadzu XRD-6000 diffractometer (Shimadzu, Columbia, USA) operated at 40 kV and 30 mA with Cu Kα radiation (1.5406 Å).

The X-ray source for EDXRF analysis was a Philips Mo X-ray tube (Philips Co., Amsterdam, The Netherlands), which irradiated the samples rectangularly with secondary Mo radiation. The semiconductor SiLi (Canberra Packard, Vienna, Austria) was used. The working parameters for irradiating the samples were 45 kV and 35 mA in a vacuum at 100 bar for 100 s. The number of pulses of the characteristic Zr-Kα irradiation was analysed with the IAEA QXAS software. 

The results are shown in the form of tables and graphs. Quantitative data are presented in the form of range, arithmetic mean and standard deviation. In the case of a non-parametric distribution, these data are presented with medians and interquartile ranges. The Shapiro–Wilk test was used to test the fit of the data to the normal distribution. Levene’s test was used to check the homogeneity of the variances. The ANOVA test and the Tukey test for multiple comparisons were used to compare the feature values between different treatments of the sample surface. Box and Whiskers plots were used for graphical representations of the results. Surface roughness after treatments was described by the mean, standard deviation, median, first and third quartile, minimum and maximum. The software used for the analysis was Statistical Analysis Software (SAS) on the Windows platform.

## 3. Results

### 3.1. Surface Roughness

**Figure 1:**
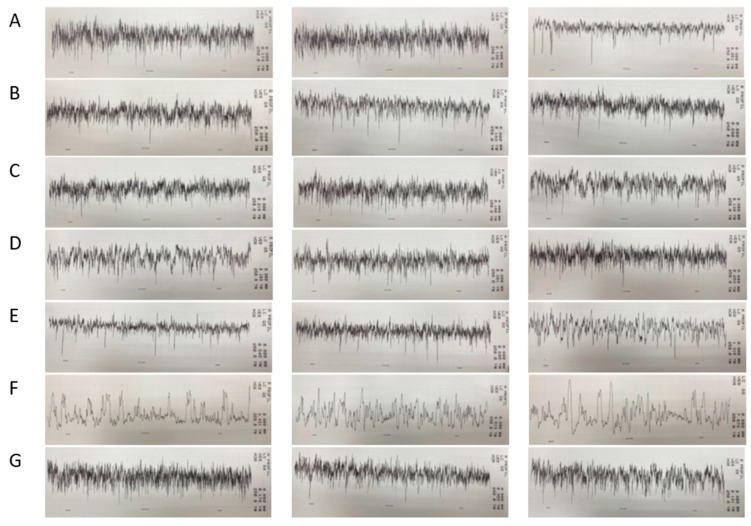
Surface roughness profiles for the first, third and fifth sample within each group (**A**–**G**).

The surface roughness profiles of the samples representative of each group are shown graphically in Figure 1. The height of the z-scale of group G is 10 times smaller than the z-scale of the other groups.

The Shapiro–Wilk test shows that the data distribution for the two roughness data sets Ra (arithmetic average roughness value) and Rz (mean roughness depth) does not deviate significantly from the normal distribution (Table 2 and Table 3, *p* > 0.05). Levene’s test shows significant inhomogeneity of variances (*p*(Rz) = 0.0165; *p*(Rz) = 0.0173). The data were logarithmised to compensate for the variances. With logarithmised data, the variability of the standard deviation is lower. According to the Levene test for logarithmised data, the homogeneity of the variances was confirmed (*p*(Ra) = 0.08 i *p*(Rz) = 0.27).

**Figure 2:**
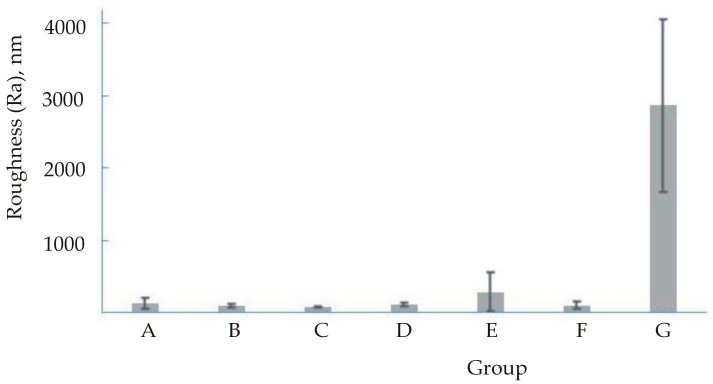
Mean value and standard deviation for roughness (Ra).

**Figure 3:**
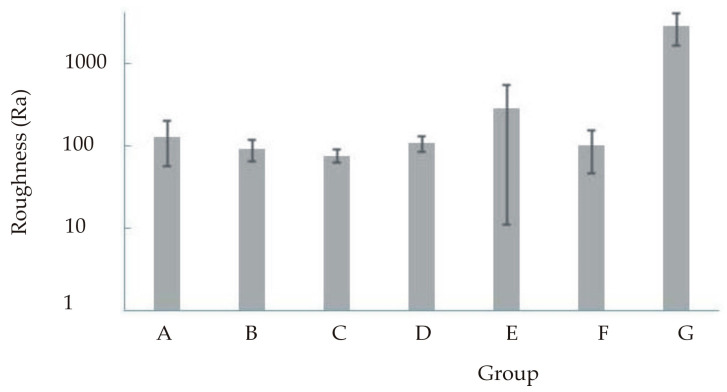
Mean value and standard deviation (logarithmised) for roughness (Ra).

**Figure 4:**
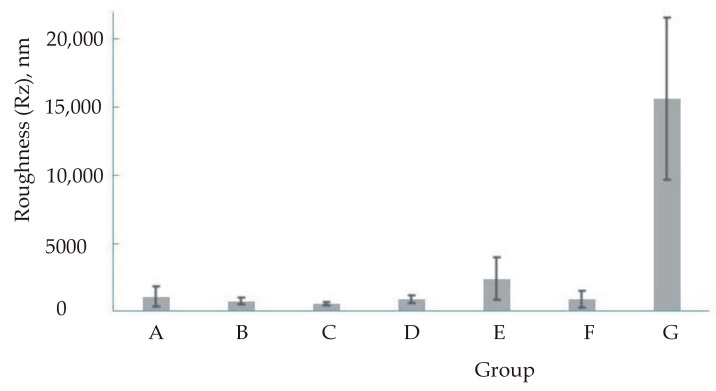
Mean value and standard deviation for roughness (Rz).

**Figure 5:**
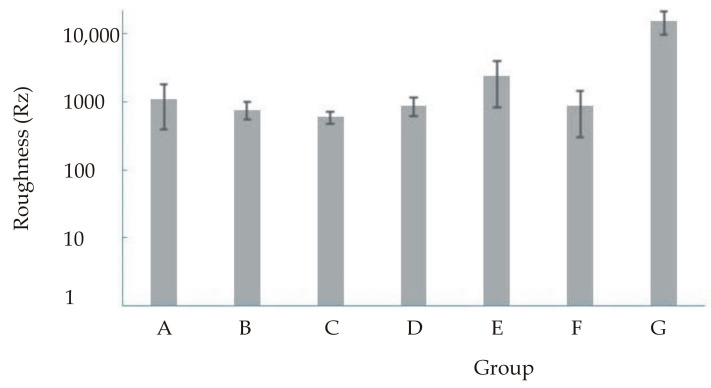
Mean value and standard deviation (logarithmised) for roughness (Rz).

Descriptive statistics for roughness are presented in Table 4 and Table 5 and Figure 2, Figure 3, Figure 4 and Figure 5. Figure 3 and Figure 5 show a logarithmic presentation of the data from Figure 2 and Figure 4 to increase clarity.

The ANOVA test shows that there is a difference in roughness for the different samples tested, both for Ra and Rz (*p* < 0.0001). The Tukey’s post hoc test comparing the samples showed that the roughness of the combination of Er:YAG laser and silanisation is higher than the roughness of the other six samples (both Ra and Rz) where no difference in roughness was found.

### 3.2. XRD Analysis

**Figure 6:**
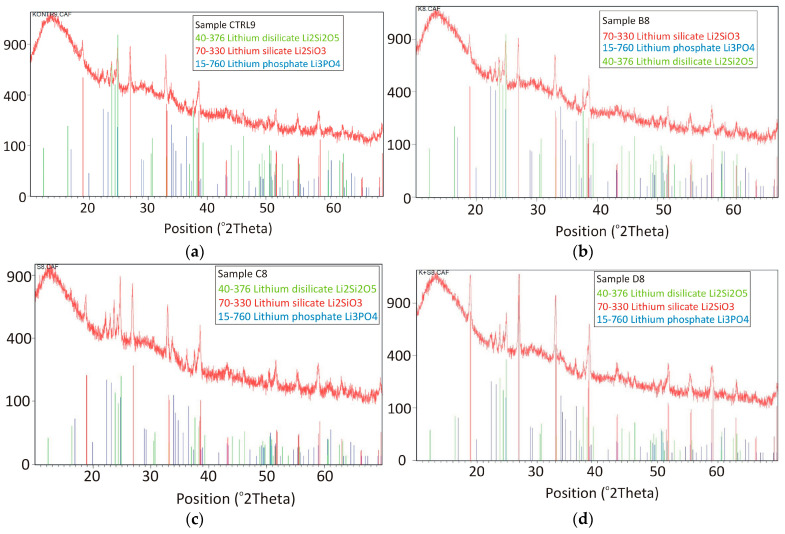

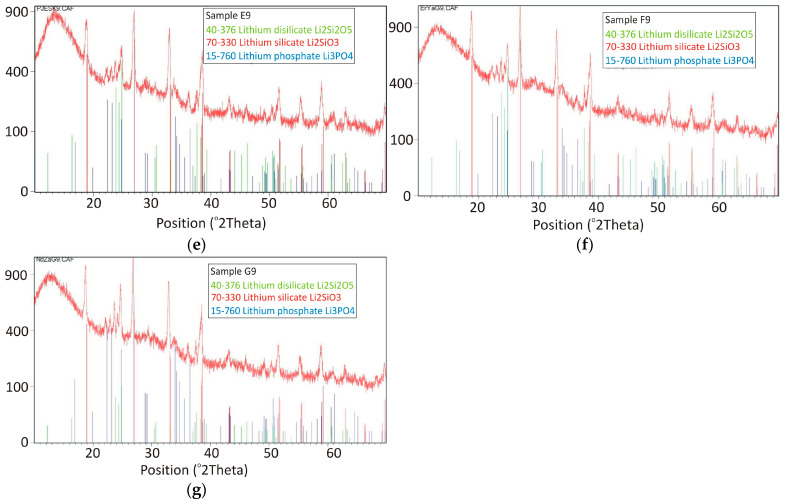
Graphical presentation of the X-ray diffraction analysis: (**a**) Group A; (**b**) Group B; (**c**) Group C; (**d**) Group D; (**e**) Group E; (**f**) Group F; (**g**) Group G.

The mineral composition of all samples was determined by XRD (X-ray diffraction) analysis. The XRD analysis of samples are shown graphically in Figure 6a–g. 

The XRD profiles of all samples show crystallisation peaks corresponding to lithium disilicate (Li_2_Si_2_O_5_), lithium silicate (Li_2_SiO_3_) and lithium phosphate (Li_3_PO_4_), indicating the existence of lithium disilicate, lithium silicate and lithium phosphate. It is obvious that the different procedures of surface treatment had no influence on the mineral composition of the surface. 

### 3.3. EDXRF Analysis

EDXRF (energy dispersive X-ray fluorescence) analysis was used to determine whether the elemental composition of the sample changes as a function of surface treatment. The change is present when the number of Zr-Kα pulses changes. The results of the EDXRF analysis are shown in Table 6.

**Figure 7:**
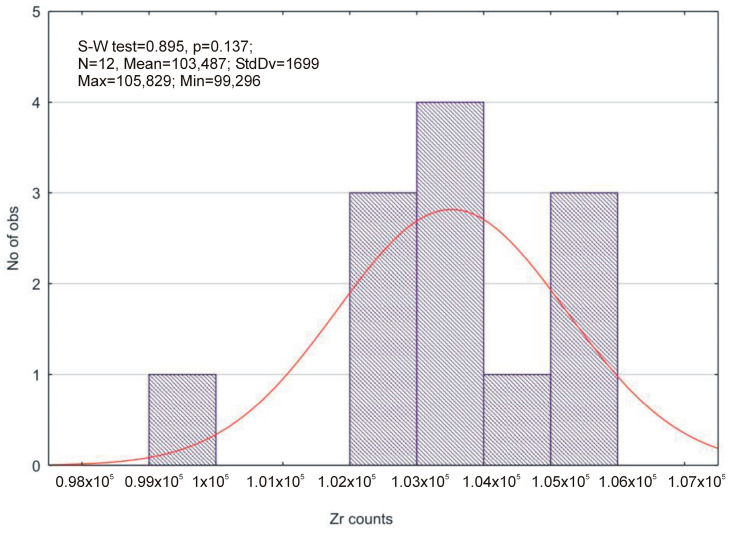
Results of the Shapiro–Wilk test and descriptive statistics.

Using the Shapiro–Wilk test (Figure 7), it was found that the measurements correspond to a normal distribution (*p* > 0.05), i.e., none of the applied surface treatment procedures change the number of pulses for Zr-Kα in the investigated sample.

**Figure 8:**
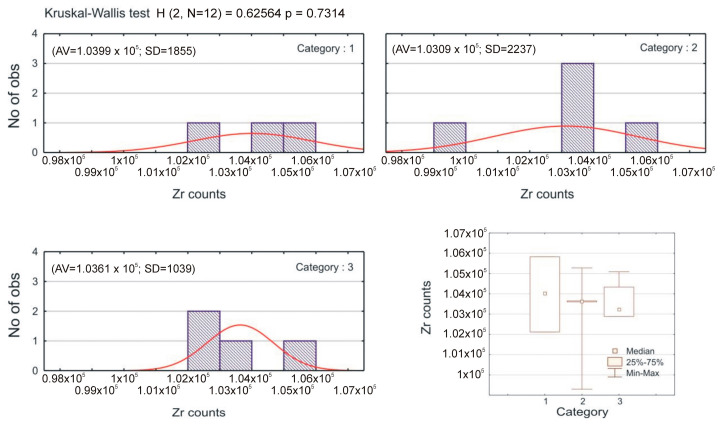
Results of the Kruskal–Wallis test and box-plot diagram by category.

The surface treatment methods were divided into categories used in the Kruskal–Wallis test (Figure 8). 

Category 1 represents the control group, category 2 includes conventional methods, while category 3 represents laser irradiation. The results of the Kruskal–Wallis test show that the medians between these three categories do not differ statistically significantly (*p* > 0.05).

## 4. Discussion

Due to the increasing demands of patients, dental ceramics are being used more and more frequently in fixed prosthetics. In addition to their excellent aesthetics, ceramics also have good biomechanical properties that make it possible to combine aesthetics and the function of the stomatognathic system. 

One type of ceramic commonly used in fixed prosthetics is glass-ceramic due to its aesthetic and mechanical properties [20], wear resistance [21], biocompatibility [22] and low thermal conductivity [20]. To ensure the durability and favourable biomechanics of ceramic work, the ceramic surface must be treated with chemical agents before cementation. As technology has evolved, so has the effect of dental lasers on the roughness of the ceramic surface. The dental laser for the preparation of the surface of prosthetic works is considered a new procedure that changes the microstructure of the surfaces and is easy to control [23,24]. Previous research has shown that different types of lasers with certain parameters release energy that has a positive effect on surface roughness by creating micro cracks that provide an additional retention surface and better bond strength. However, Ural and Kalyoncuglu demonstrated that laser energy can also reduce the quality of the bond by melting the surface of the ceramic, smoothing the surface and reducing the bond strength [25]. On the other hand, Akin et al. showed that the Er:YAG (erbium-doped yttrium aluminium garnet laser) laser creates microcracks and additional surface retentions, thus improving the bond strength [26]. From these studies, it can be concluded that the effects of dental lasers on the ceramic surface and bond strength are highly unpredictable due to too many variables (type of ceramic, parameters of the laser, duration of treatment) that need to be researched and standardised.

To ensure the bond between the ceramic prosthetic work and the abutment tooth, both the surface of the tooth and the inner surface of the restorations must be prepared. To achieve optimal micromechanical retention, the inner surface of the restorations must be conditioned, which creates microporosity and increases the contact surface and thus the mechanical retention of the cement. Surface treatments such as abrasion, sandblasting and acid etching to increase roughness and micromechanical retention are well described in the literature [27,28]. All of the above procedures have been tested in in vitro studies, which must be viewed with caution due to their limitations. The stability of materials for use in dental medicine is important to maintain biocompatibility under the complex conditions of the oral cavity. Biocompatibility implies a balance between function, the materials used and the host. The evaluation of the biocompatibility of materials involves several types of biological tests, physical property tests and risk-benefit analyses. All tests must be standardised and reproducible. Test methods and definitions of mechanical properties of strength and hardness, testing of friction, surface roughness and adhesion of materials are performed in vitro. Surface roughness parameters describe unevenness on the surface of the material related to the production method, office handling and corrosion. Depending on the magnitude, they are measured with a profilometer or with an atomic force microscope (AFM). The testing of materials used in dental medicine must be comprehensive and continuous. In addition to the general requirements that must be met, each material also has specific requirements that depend on the function it performs, the site of application and the site of contact with the surrounding tissue. Testing must be performed according to internationally recognised standards and any clinically observed changes in the weakening of the material’s properties and function, as well as patient responses, must be recorded, renewed and replaced to maintain the material’s biocompatibility. New technologies, scientific progress and commercial viability allow for the continuous development of new materials and procedures in dentistry, the independent evaluation of which by standardised tests is necessary and constantly required [29]. Nevertheless, the results can be interpreted with a high degree of certainty as developments in the oral cavity. In addition, in vitro studies are easier, cheaper and faster to perform.

Tian reported that hydrofluoric acid etching and silanisation is the most commonly used treatment prior to the cementation of glass-ceramic restorations [30]. This pre-treatment resulted in a partially dissolved surface and partially exposed crystals, which roughen the surface of the ceramic and contribute to micromechanical retention with the resin cement. An additional increase in roughness increases the surface energy and the interaction between the binder and the silane [31]. The main role of the cement is to ensure the good retention of the restoration and the quality of the marginal fit. In addition, it contributes to the optical properties in modern materials. Due to the aesthetic properties of composite cements, they are increasingly used in dental medicine. The composite cement consists of three main components: the organic resin matrix of bisphenol A-glycidyl methacrylate (Bis-GMA) or urethane dimethacrylates (UDMA), inorganic filler particles and a cross-linking bonding agent intermediate layer. In addition to high compressive and tensile strength, composite cements have the ability to create a micromechanical bond with enamel, dentin, dental alloys and ceramics. Composite cements, in combination with an adhesive, create a mechanical, micromechanical and chemical bond between the two materials so that primary retention is not required. For the quality of the adhesive bond, it is important to prepare the surfaces of the tooth and the fixed prosthetic replacement. In current clinical practise, ceramics are etched, sandblasted or a primer may be added. Etched glass-ceramics can be cemented with adhesive cements because the glassy surface layer has been removed. There is no agreed opinion on the treatment method of the restoration surface, but the recommended treatments are the roughening of the surface, chemical bonding and laser treatment. In this study, all available methods (etching, sandblasting, silanisation and their combinations) are tested together with the dental lasers. The clinical success of prosthetic therapy with all ceramic restorations depends on the quality of the bond between the prosthetic appliance and the bonding agent and the formation of the monoblock with the structures of the oral cavity. The bond is established by micromechanical and chemical retention. Micromechanical retention is achieved by etching with hydrofluoric acid and sandblasting, while silanisation ensures chemical retention. 

In this study, the surface roughness of lithium disilicate glass-ceramic reinforced with zirconia was measured using standard profilometry. For this purpose, seventy samples were produced and divided into seven groups depending on the surface treatment (A—control group without treatment, B—etching with 9.5% hydrofluoric acid, C—silanisation, D—etching with 9.5% hydrofluoric acid and silanisation, E—sandblasting and silanisation, F—Er:YAG laser irradiation and silanisation, G—Nd:YAG (neodymium-doped yttrium aluminum garnet) laser irradiation and silanisation). The highest surface roughness was obtained by combining Er:YAG and silanisation while the lowest values of surface roughness were found in the samples treated with silane. The values of surface roughness after treatment with both lasers (Er:YAG and Nd:YAG), etching, sandblasting, and silanisation show the most statistically significant, highest value with the Er:YAG laser. The hydrofluoric acid weakened the surface, as shown by the mean value of roughness. This is also confirmed by other authors [32]. The glass and crystals of glass-ceramics are extremely damaged by sandblasting. Ustun et al. claim that the surface treatment affects the surface roughness and state that higher bond strength values are obtained by sandblasting than by Er:YAG laser [33]. The results of this work showed the second highest values of surface roughness for the samples that were sandblasted and then silanised. All of the above treatments require a micromechanical interlock on the bonding surface and a chemical bond between the bonding surfaces, which means that the texture of the surface of the material or tooth must be interfered with. When the texture and chemical properties of the surface are changed, the surface appears more active and functional. [34]. The acid dissolves the surface of the ceramic by dissolving the glass phase, which causes irregularities on the surface, increasing the contact area. [35]. The physiochemical interaction between composites and ceramics leads to their adhesion, which is achieved by the surface treatment and its topography. Sandblasting changes the topography and moisture of the surface, which correlate with the surface energy and adhesion potential [36]. The architecture of the surface is visible at the micro level, which is important for conducting research with sophisticated equipment. Mechanical retention increases with increasing surface roughness due to adhesive interlocking between surface irregularities. [37]. On the other hand, several studies have shown that there is a possibility of fracture of the restoration due to the weakening of the ceramic surface after etching [38]. Although the use of the laser for surface treatment before cementation has its difficulties, it is nevertheless promising. A 10.6 μm CW CO_2_ (carbon dioxide) laser was tested on lithium disilicate [39] and CAD/CAM (computer-aided design/computer-aided manufacturing) ceramics [40] confirming the presence of microfractures and surface dissolution as a result of the thermal effect of laser irradiation at a power greater than 10 W CW (3184.7 W/cm^2^) [39,40]. However, examination of the ceramic structure after irradiation with a pulsed Nd:YAG laser at 10 W (14.185 W/cm^2^) and 1340 nm reveals the presence of channels, micro cracks and dissolved crystals. These changes are probably the result of enormous energy accumulation due to the high quantum radiation energy concentrated on a specific area over a short period of time. High thermal values generated by CO_2_ and Nd:YAG laser irradiation lead to extreme physical stresses and additional hardening of the ceramic surface, which can cause the micro cracks mentioned. [25,41]. Although the Er:YAG laser can be used to treat feldspathic ceramics, the result obtained by etching is much stronger. The reason for this could be that the energy generated by the Er:YAG laser cannot be absorbed well in this type of ceramic, so that the micromechanical retention is not sufficient [42]. To achieve adequate retention, some authors recommend the use of a very high energy (500 mJ) [43]. Better results could be achieved with new ultra-short pulsed lasers [44]. Despite numerous studies, laser radiation is still an alternative surface treatment method for a better bond between two contact surfaces. Laser radiation does not produce the required roughness of ZrO_2_ (zirconium dioxide, zirconia) ceramics. The irregularities are too small to provide a micromechanical hold, so the bond strength does not increase. Comparing the laser and tribochemical treatment methods, tribochemical treatment is more efficient than the laser [45]. Based on SEM (scanning electron microscopy) analysis [6], it is assumed that the surface is still rough after treatment. It also contains homogeneous round microretention and shallow holes, but no micro-cracks [45]. Silanisation allows the infiltration of the composite into the irregularities of the ceramic surface, which causes a chemical bond of the silane with the molecules of the composite, creating a siloxane network. This results in better contact and the infiltration of the composite into the irregularities of the ceramic, better protection against moisture and the creation of an acidic environment that can support the bonding mechanisms [46]. Bonded indirect restorations with different internal surface roughness obtained with the methods described shall be tested with aging simulations [47] and cyclic fatigue [48] to better simulate clinical scenarios. Limitations of this study include the fact that the research was conducted in vitro and it is not known how the oral cavity and human body would actually respond to implanted ZLS treated with the procedures described previously. For this reason, future work should simulate in vivo conditions and conduct clinical trials.

## 5. Conclusion

The aim of this study was to investigate the influence of different methods of surface treatment of lithium disilicate glass-ceramics reinforced with zirconia on the surface properties and surface roughness for bonding with self-adhesive resin cement. The following conclusions can be drawn from the results obtained:

- Among the samples treated with conventional procedures, the surface roughness is highest in the samples treated with sandblasting and silanisation (group E). When comparing the surface roughness of the samples treated with lasers, it is clear from the results obtained that a higher surface roughness was achieved with the Er:YAG laser (group G).
- The surface roughness of lithium disilicate reinforced with zirconia is the highest in samples treated by Er:YAG irradiation and silanisation,
- The analysis of the ceramic surface by means of EDXRF analysis and XRD analysis shows that none of the applied surface treatment procedures change the composition of the surface.

## Figures and Tables

**Table 1:** Groups based on surface treatments.

Group	Surface Treatment
A	Control group
B	9.5% hydrofluoric acid (HF)
C	Silanisation
D	9.5% hydrofluoric acid (HF) and silanisation
E	Sandblasting and silanisation
F	Nd:YAG irradiation and silanisation
G	Er:YAG irradiation and silanisation

**Table 2:** Test of normality of surface roughness data (Ra).

Group	Surface Roughness (Ra), nm					Logarithmised Data				
						Surface Roughness (Ra)				
	N	Mean Val.	St. Dev.	W **	*p* *	N	Mean Val.	St. Dev.	W **	*p* *
A	3	127.8	71.8	0.9	0.4	3	4.75	0.54	0.96	0.61
B	3	90.2	26.7	0.86	0.26	3	4.47	0.33	0.84	0.2
C	3	75.2	13.9	0.8	0.1	3	4.31	0.2	0.79	0.09
D	3	106.8	22.8	0.94	0.54	3	4.66	0.21	0.96	0.63
E	3	282.1	271.2	0.84	0.21	3	5.34	0.94	0.93	0.48
F	3	100.7	54.7	0.9	0.37	3	4.52	0.52	0.95	0.57
G	3	2863.3	1194.5	1	0.92	3	7.89	0.46	0.97	0.69

* *p*-value for Shapiro–Wilk test; ** W—statistics value for Shapiro–Wilk test.

**Table 3:** Test of normality of surface roughness data (Rz).

Group	Surface Roughness (Rz), nm					Logarithmised Data				
						Surface Roughness (Rz)				
	N	Mean Val.	St. Dev.	W **	*p* *	N	Mean Val.	St. Dev.	W **	*p* *
A	3	1095.4	701.1	0.88	0.32	3	6.87	0.61	0.94	0.54
B	3	763.1	223.4	0.78	0.07	3	6.60	0.33	0.77	0.06
C	3	592.7	114.1	0.87	0.29	3	6.37	0.20	0.85	0.24
D	3	879.3	269.1	0.95	0.56	3	6.75	0.30	0.98	0.70
E	3	2382.7	1557.8	0.93	0.48	3	7.63	0.65	0.99	0.78
F	3	887.9	583.6	0.86	0.27	3	6.65	0.62	0.92	0.47
G	3	15592.2	5958.0	1.00	0.95	3	9.60	0.40	0.99	0.84

* *p*-value for Shapiro–Wilk test; ** W—statistics value for Shapiro–Wilk test.

**Table 4:** Comparison of the roughness (Ra) between groups.

Group	Surface Roughness (Ra), nm							ANOVA
	Mean Val.	St. Dev.	Median	Q1	Q3	Min.	Max.	*p*
A	127.8	71.8	102.0	72.5	209.0	72.5	209.0 ^a^	<0.0001
B	90.2	26.7	101.8	59.7	109.2	59.7	109.2 ^a^	
C	75.2	13.9	82.5	59.2	84.0	59.2	84.0 ^a^	
D	106.8	22.8	100.5	87.8	132.2	87.8	132.2 ^a^	
E	282.1	271.2	155.5	97.3	593.5	97.3	593.5 ^a^	
F	100.7	54.7	80.3	59.2	162.7	59.2	162.7 ^a^	
G	2863.3	1194.5	2923.3	1640.0	4026.7	1640.0	4026.7	

^a^ ‘post hoc’ test, the same letter denotes materials that differ from each other.

**Table 5:** Comparison of the roughness (Rz) between groups.

Group	Surface Roughness (Rz), nm							ANOVA
	Mean Val.	St. Dev.	Median	Q1	Q3	Min.	Max.	*p*
A	1095.4	701.1	813.2	579.5	1893.7	579.5	1893.7 ^a^	<0.0001
B	763.1	223.4	883.5	505.3	900.5	505.3	900.5 ^a^	
C	592.7	114.1	640.7	462.5	675.0	462.5	675.0 ^a^	
D	879.3	269.1	809.0	652.3	1176.5	652.3	1176.5 ^a^	
E	2382.7	1557.8	1897.2	1125.5	4125.5	1125.5	4125.5 ^a^	
F	887.9	583.6	637.3	471.5	1555.0	471.5	1555.0 ^a^	
G	15592.2	5958.0	15405.0	9730.0	21641.7	9730.0	21641.7	

^a^ ‘post hoc’ test, the same letter denotes materials that differ from each other.

**Table 6:** Results of energy dispersive X-ray fluorescence analysis.

ID Spectrum	Group	Number of Zr-Kα Pulses	Category
VC8872	A	104027	1
VC8860	B	103649	2
VC8861	C	99296	2
VC8862	D	103601	2
VC8863	E	103620	2
VC8866	F	105084	3
VC8867	G	102882	3

## Data Availability

Not applicable.
